# Potential primary roles of glial cells in the mechanisms of psychiatric disorders

**DOI:** 10.3389/fncel.2015.00154

**Published:** 2015-05-15

**Authors:** Kazuhiko Yamamuro, Sohei Kimoto, Kenneth M. Rosen, Toshifumi Kishimoto, Manabu Makinodan

**Affiliations:** ^1^Department of Psychiatry, Faculty of Medicine, Nara Medical University, KashiharaJapan; ^2^BioAxone BioSciences Inc., Cambridge, MAUSA

**Keywords:** glia, schizophrenia, autism, mouse models, astrocytes, oligodenrocytes, microglia, MeCP2

## Abstract

While neurons have long been considered the major player in multiple brain functions such as perception, emotion, and memory, glial cells have been relegated to a far lesser position, acting as merely a “glue” to support neurons. Multiple lines of recent evidence, however, have revealed that glial cells such as oligodendrocytes, astrocytes, and microglia, substantially impact on neuronal function and activities and are significantly involved in the underlying pathobiology of psychiatric disorders. Indeed, a growing body of evidence indicates that glial cells interact extensively with neurons both chemically (e.g., through neurotransmitters, neurotrophic factors, and cytokines) and physically (e.g., through gap junctions), supporting a role for these cells as likely significant modifiers not only of neural function in brain development but also disease pathobiology. Since questions have lingered as to whether glial dysfunction plays a primary role in the biology of neuropsychiatric disorders or a role related solely to their support of neuronal physiology in these diseases, informative and predictive animal models have been developed over the last decade. In this article, we review recent findings uncovered using glia-specific genetically modified mice with which we can evaluate both the causation of glia dysfunction and its potential role in neuropsychiatric disorders such as autism and schizophrenia.

## Introduction

Glial cells are the non-excitable supporting cells of the central nervous system (CNS) and classified mainly as oligodendrocytes, astrocytes, and microglia. These cells are typically smaller in size, but can be far more numerous than neurons in certain brain regions such as the cerebral cortex. Overall, the ratio between neurons, and glial cells in the human CNS is approximately 4:1 (Azevedo et al., 2009), with oligodendrocytes being the most abundant type of glial cells (75.6%), followed by astrocytes (17.3%) and microglia (6.5%) in human male brains (Pelvig et al., 2008). Glial cells clearly provide “support" for both cells such as neurons and structures such as blood vessels, but also can function to increase action potential conduction velocity via saltatory conduction from one node of Ranvier to the next in myelinated axons, and also the response to damage in the CNS via gliosis, a non-specific reactive change in glial cells associated with their proliferation or hypertrophy. Convergent lines of evidence from multiple studies in neuroimaging, postmortem brains, and genome-wide association studies (GWAS) have revealed a wide range of white matter abnormalities in schizophrenia (Dwork et al., 2007; Bernstein et al., 2015). Indeed, the implication of oligodendrocytes and myelin in schizophrenia has come from analyses of postmortem brains using microarray gene expression (Iwamoto et al., 2005; Katsel et al., 2005), protein expression (Dracheva et al., 2006), electron microscopic studies (Uranova et al., 2011), and neuroimaging (Kubicki et al., 2007).

Astrocytes are regarded as neuronal partners since they hold concerted cross-talk with neighboring neurons, which is crucial for normal brain function. Astrocytes are able to sense neurogenesis, development, and maturation of brain circuits and neuronal activity leading to both homeostatic changes and increased cellular crosstalk (Wang and Bordey, 2008; Parpura et al., 2012). The homeostatic responses of astrocytes includes increases in metabolic activity, the synthesis of a neuronal preferred energy substrate lactate, clearance of neurotransmitters and buffering of extracellular K^+^ ions to name but a few (Kimelberg, 2007; Wang and Bordey, 2008; Parpura and Verkhratsky, 2012). The existence of bidirectional communication between astrocytes and neurons during synaptic communication and function has been conceptualized as the “tripartite synapse” with its associated alterations in neuron–glia cross talk (Newman, 2003; Perea et al., 2009).

Oligodendrocytes, the myelin forming cells of the CNS, have a small round cell body and about 4–6 branching processes, which can myelinate up to 60 axons depending on the diameter (Miller, 2002). By ensheathing axons, mature oligodendrocytes provide critical insulation to facilitate axonal conduction by increasing the resistance and reducing the effective capacitance of the axonal membrane, resulting in faster conduction speed in myelinated axons compared to unmyelinated axons of the same diameter. In addition, recent studies have provided unique roles of oligodendrocytes, indicating that myelination of the axons cannot only influence neuronal properties in ways not previously considered, but also may be a key source of trophic and metabolic support for maintaining axonal integrity (Nave, 2010).

Microglia function as not only members of the innate immune system but also participate in synaptic modulation and maturation, learning, and memory processes. Importantly, they extend a broad network of ramified processes in the CNS parenchyma. After an injury to the brain, microglia rapidly extend highly active exploratory processes into the sites of injury without any corresponding cell body movement, potentially establishing a barrier between healthy and injured tissues in which microglia actively and constantly interact with neurons and astrocytes and survey the local environment (Davalos et al., 2005; Nimmerjahn et al., 2005). Moreover, microglia can directly regulate both synaptic function and synaptic maintenance in the absence of injury or neuroinflammation (Bessis et al., 2007; Wake et al., 2009).

The focus of this review is to examine what is known regarding the relationship between altered glial cell function and the pathobiology of psychiatric disorders, and whether glial dysfunction can play a causative role. First, we examine the monogenic disorder Rett syndrome (RTT) and examine the mouse model harboring a mutant *methyl-CpG binding protein 2* (MeCP2) gene where neuronal and glial biology have been extensively investigated (**Table 1**). Subsequently, we examine several other mouse models for schizophrenia (**Table 2**) and how glial cell function or dysfunction contributes both to the phenotype and pathophysiology.

**Table 1:** Summary of mutant MeCP2 mouse models.

Genotype	Phenotype	Reference
**MeCP2**
**MeCP2-deficiency astrocytes** *MeCP2tm1.1Bird/+ mice*	Reduced BDNF Reduced IL-1β and IL-6 Hyper-activated p38MARK pathways Abnormal neuronal dendritic neurodevelopment	Maezawa et al. (2009)
**MeCP2-deficiency astrocyte *in vitro* co-culture**	MeCP2 is present in all glial cell types in normal brain Abnormal neuronal dendritic neurodevelopment	Ballas et al. (2009)
**In globally MecP2 deficit mice, re-expression of MeCP2 preferentially in astrocytes**	Improved locomotion and anxiety level Restored respiratory abnormalities	Lioy et al. (2011)
**MeCP2-deficiency microglia** *MeCP2tm1.1Bird/+ mice*	Increased glutamate release Increased neurotoxicity Glutamate inhibitor, gap channel hemichannel blocker, and glutamate receptor antagonist blocked the neurotoxicity	Maezawa and Jin (2010)
**Re-expression of MeCP2 in microglia in MeCP2 null mice**	Improved RTT-like phenotype	Derecki et al. (2012)
*MeCP2tm1.1Bird/+ and MecP2tm1.1Jae mice*	Related phagocytic activity	
**Re-expression of MeCP2 in oligodendrocytes in MeCP2 null mice**	Milder RTT-like phenotype	Nguyen et al. (2013)
*MeCP2loxJ/y/NRG2Cre mice*	Mildly prolonging their life span	
	Significantly improved the locomotor deficits and hindlimb clasping both in male and female	
	Fully restored the body weight	
	PLP and MBP remain reducing	

**Table 2:** Summary of mutant DISC1, neuregulin-ErbB signaling mouse models, and others.

Genotype	Phenotype	Reference
**DISC1**
**DISC1-deficiency astrocyte**	Diminished production of D-serine	Ma et al. (2013)
*B6.Cg-Tg(GFAP-tTA) 110Pop/J mice*	Abnormal behavior similar to schizophrenia	
	Increased sensitivity to NMDA antagonist and he ameliorative effects of D-serine	
**DISC1-increased or decreased expression oligodendrocyte**	Knockdown of DISC1 increased oligodendrocytes differentiation DISC1 overexpression decreased oligodendrocytes differentiation	Hattori et al. (2014)
**DBZ-decreased expression oligodendrocyte** *DBZ KO mice*	Myelination in the corpus callosum was delayed but is mostly recovered by adulthood Oligodendrocytes with immature structural features were more abundant	Shimizu et al. (2014)
**Neuregulin-ErbB signaling**
**Astrocyte specific disruption of synCAM1 signaling**	Increased locomotor activity in the dark period that are attenuated by the psychostimulant D,L-amphetamine	Sandau et al. (2012)
*GFAP-DNSynCAM1 mice*	Exhibited attenuation of changes in diurnal rhythm activity	
	Reduced anxiety levels	
	Reduced the acoustic startle paradigm	
**Neuregulin-ErbB4 receptor signaling-deficiency oligodendrocyte**	Myelin sheath is significantly thinner and OLs morphology is less complex	Roy et al. (2007)
*CNP-DNErbB4 mice*	Heightened anxiety and had an abnormal behavior	
	Enhanced sensitization to amphetamine	
	Altered dopamine signaling including levels of DAT and D1-like receptor binding	
**Neuregulin-ErbB3 receptor Signaling-deficiency oligodendrocyte** *PLP/CreERT-ErbB3flox/flox mice*	Deletion of selective ErbB3 receptor in oligodendrocytes Deficits in social interaction and working memory	Makinodan et al. (2012)
**Others**
**Mice with proteolipid protein overexpression** *plpl^tg/-^ mice*	The myelin was intact at the 2 months of age, but developed demyelination Reduced conduction velocity	Tanaka et al. (2009)
	Increased anxiety	
	Spatial learning deficits and working memory deficits	
**Nogo-A knock out mice**	Sensorimotor deficit	Willi et al. (2010)
*Nogo-A^-/-^ mice*	Motor stimulant response to systemic amphetamine	
	Increased dopamine D2 receptor expression in the striatal and limbic regions	
**Selective overexpression of heme oxygenase-1 (HO-1) in astrocytes**	Hyperdopaminergic tone in the nigrostriatal and mesolimbic systems	Song et al. (2012)
*HMOX1 transgenic mice*	Sensorimotor deficit	

## MeCP2

Rett syndrome is currently considered a severe neurodevelopmental disorder caused by sporadic mutations in the *X-chromosome-linked gene* MeCP2 (Amir et al., 1999). Females born with RTT develop normally for 6–18 months and then begin to regress, losing speech, motor skills, and purposeful hand motions and suffering other severe problems including mental retardation, epileptic seizures, and overall retarded growth (Hagberg, 2002). In fact, RTT brain shares certain features with regressive type autism including small neuronal size, as well as reduced dendritic branching and spines in selected regions (Zoghbi, 2003; Armstrong, 2005).

### MeCP2 Knockout Mice

In fact, male MeCP2 null mice show severe neurological symptoms at approximately 6 weeks of age, while heterozygous female mice also develop behavioral symptoms after several months (Guy et al., 2001). Loss of MeCP2 function in RTT mice leads to abnormalities in dendritic arborization (Armstrong, 2005), basal synaptic transmission (Moretti et al., 2006), excitatory synaptic plasticity (Asaka et al., 2006; Moretti et al., 2006; Chao et al., 2007) and reduced spontaneous cortical activity (Dani et al., 2005). Studies utilizing a mouse line with a conditional MeCP2 gene knockout specific to neural stem/progenitor cells, (*nestin*-Cre/*MeCP2*-/y) identified phenotypes that resemble some of the symptoms of RTT-like phenotypes (Chen et al., 2001; Guy et al., 2001). However, mice carrying a conditional knockout of MeCP2 in post-mitotic neurons driven by the *calcium/calmodulin-dependent protein kinase 2 (Camk2)-cre* transgene (*CamkII*-Cre/*MeCP2*-/y; Chen et al., 2001) exhibit milder and delayed RTT-like phenotypes compared with the *nestin-cre* driven transgenic mice (Chen et al., 2001). While the neurological symptoms of MeCP2 knockout mice are reversed by restoring MeCP2 expression (Guy et al., 2007), normal MeCP2 expression in neuronal cells is unable to prevent the phenotypes of the MeCP2 null mice (Alvarez-Saavedra et al., 2007), which implicates the specific loss of glial MeCP2 expression in the pathobiology of RTT. Thus, while MeCP2 is widely expressed throughout various cell types in the normal brain including neurons, and all types of glial cell such as astrocytes, oligodendrocytes, and microglia (Ballas et al., 2009), it appears plausible that while neuronal dysfunction was formerly viewed as a significant contributor to RTT causation, glial dysfunction actually may play a greater role in the development of RTT.

### MeCP2-Deficiency in Astrocytes

With the loss of MeCP2 expression in astrocytes, there are significant abnormalities in the expression of *brain-derived neurotrophic factor* (BDNF); an established target of MeCP2 binding (Chang et al., 2006). Interestingly, astrocytes are known to be involved in the initiation and regulation of nervous system immune responses through the release of proinflammatory cytokines (Farina et al., 2007), and it is noteworthy that the expression of interleukin (IL)-1β and IL-6 in response to administration of lipopolysaccharide is reduced in this model compared to that of controls. In addition, *p38 mitogen activated protein kinases* (MARK) pathways are hyper-activated in this model irrespective of exposure to lipopolysaccharide.

A prominent neuropathological feature associated with brains of RTT is small neuronal size and reduction in dendritic branching and spine density (Armstrong, 2005). Since neurons with more extensive contact with astrocytes promote more extensive dendritic growth (van den Pol and Spencer, 2000), co-culture experiments using intact neurons and astrocytes with MeCP2 deficiency were performed. In this study, neurons cultured in the presence of the MeCP2 deficient astrocytes displayed a much less developed dendritic arborization than did neurons cultured with wild type astrocytes (Ballas et al., 2009). Furthermore, the engineered re-expression of MeCP2 in astrocytes *in vivo* mouse model led to both significantly improved locomotion and anxiety levels as well as respiratory state (Maezawa et al., 2009; Lioy et al., 2011).

### MeCP2 Deficiency in Oligodendrocytes

When mice were engineered that lacked MeCP2 expression in oligodendrocytes, they showed a normal lifespan and the symptoms associated with the RTT-like phenotype commenced at ~10 weeks of age and were milder than those of MeCP2 null mice where the symptoms typically began at 4–5 weeks of age (Chen et al., 2001). With the observational phenotypic scoring system (score = 0–10) considering five typical RTT phenotypic traits such as mobility, gait, hindlimb clasping, tremors, and general conditions (Guy et al., 2007; Nguyen et al., 2012), while MeCP2 null mice reached a score of 6–10 between 9 and 15 weeks of age (Chen et al., 2001), oligodendrocyte MeCP2 knockout mice reached a score of 2 at 20 weeks of age. Additionally, these mice are more active and develop severe hindlimb clasping phenotypes. Restoration of MeCP2 expression solely in cells of the oligodendrocyte lineage in MeCP2 global null mice partially reverses the RTT-like phenotypes associated with loss of MeCP2 such as diminished life span, locomotor deficits, and hindlimb clasping both in male and female, and fully restored normal body weight. However, while MeCP2 expression in the oligodendrocyte lineage cells partially rescues the aberrant expression of MBP protein, it does not affect the expression of either *2′,3′-cyclic-nucleotide-3′-phosphodiesterase* (CNPase), *myelin oligodendrocyte glycoprotein* (MOG), or *myelin proteolipid protein* (PLP) (Nguyen et al., 2012).

### MeCP2-Deficiency in Microglia

Activated microglia release a large amount of glutamate and this microglial-associated neurotoxicity is mediated primarily by NMDA receptor signaling (Takeuchi et al., 2005). In addition to glutamate, activated microglia release pro-inflammatory cytokines such as IL-1β, IL-6, interferon (IFN)-γ, and tumor necrosis factor (TNF-α), which also promote neuronal damage (Sawada et al., 1989; Mizuno et al., 1994, 2003; Suzumura et al., 1996). TNF-α secreted from activated microglia is a major neurotoxic cytokine that induces neurodegeneration through silencing of cell survival signals and caspase-dependent cascades including promotion of signaling through Fas ligand (Greig et al., 2004; Block and Hong, 2005). Neurons treated with conditioned media from MeCP2 null microglia display damaged dendrites and the concentration of glutamate in the media is five time higher than that in control media. The blocking of both microglial glutamate synthesis by a glutaminase inhibitor and microglial glutamate release by a gap junction connexin32 (Cx32) hemichannel blocker abolishes the neurotoxic activity as well as the blocking of a glutamate receptor antagonist. These reports indicate that aberrant glutaminase activity or Cx32 expression in microglia is responsible for the increased production and release of glutamate, potentially implicating these modulators in the pathobiology of microglia-induced RTT-like symptoms (Maezawa and Jin, 2010).

### Introduction of Wild Type Microglia into the MeCP2 Null Mouse

Allogeneic transplantation of wild type bone marrow into irradiation-conditioned MeCP2 null mice led to the engraftment of wild type microglia into the MeCP2 null brain parenchyma. The lifespan of these mice was significantly extended as compared with MeCP2 null mice that received either an autologous bone marrow transplant or untreated MeCP2 null mice. Similarly, body and brain weights of MeCP2-null mice recipients of wild type bone marrow recovered approximately to the level seen in wild type mice. And while the overall appearance, tremor, and gait of MeCP2 null mice recipients of wild type bone marrow were improved, the hindlimb clasping phenotype was not changed. Additionally, these mice exhibited significantly reduced numbers of apneic episodes and greatly reduced respiratory irregularities. Interestingly, these benefits resulting from engraftment with wild-type microglia were diminished when phagocytic activity was inhibited pharmacologically by using annexin V which results in substantial blocking of phagocytic activities (Lu et al., 2011; Derecki et al., 2012).

Thus, the focus of RTT studies using animal models has begun to swing from only studying potential neuronal dysfunction to include interrogation of a role for glial dysfunction and the results of the above studies lends credence to the hypothesis that glial dysfunction may play a primary rather than a secondary role in the causation of RTT-like symptoms. Similarly, studies of DISC1 knockout mice and *neuregulin 1* (NRG1) knockout mice as models for schizophrenia initially focused on the role these proteins play in neurons, but more recently, their activities in glial cells have begun to be considered.

## Disrupted in Schizophrenia 1 (DISC1)

Schizophrenia, which affects ~1% of the worldwide population, is also known as a neurodevelopmental disorder (Weinberger, 1987). The clinical features of schizophrenia cluster in three domains, characterized by positive symptoms (e.g., delusions, hallucinations, thought disorder), negative symptoms (e.g., social withdrawal, blunted affect, reduced motivation) and cognitive symptoms (e.g., attention and working memory deficits). Although schizophrenia is a complex disorder with polygenic and environmental antecedents, multiple lines of evidence have proposed some putative susceptibility genes for schizophrenia (Harrison and Weinberger, 2005; Schizophrenia Working Group of the Psychiatric Genomics, 2014). For example, two overlapping and opposite strand genes on chromosome 1, DISC1 and DISC2, are specifically disrupted by a *t*(1;11; q42.1; q14.3) balanced translocation, in a large Scottish pedigree, resulting in a cohort with several major mental illnesses such as schizophrenia, bipolar affective disorder, and recurrent major depression (St Clair et al., 1990; Millar et al., 2000, 2001; Blackwood et al., 2001; Muir et al., 2008). DISC1 is expressed in neurons within various brain areas including the olfactory bulb, cortex, hippocampus, hypothalamus, cerebellum, and brain stem, especially during development (Schurov et al., 2004). DISC1 has been shown to be involved in several neurodevelopmental processes including progenitor cell proliferation (Mao et al., 2009), radial migration (Tomita et al., 2011), dendritic arborization (Kamiya et al., 2006; Duan et al., 2007), and synapse formation (Camargo et al., 2007; Duan et al., 2007).

### DISC1 Knockout Mice

Since expression of dominant negative proteins frequently has been used successfully in animal models to achieve partial loss of function for relevant proteins (Oike et al., 1999), transgenic mice harboring a C-terminally truncated, dominant negative DISC1 (*DN-DISC1*), expressed under the control of a promoter for *αCaMKII*, were generated (Hikida et al., 2007). This model displays several abnormalities including hyperactivity, disturbance in sensorimotor gating, the dynamic modulation of reward value by effortful action, progressive ratio performance, social behavior, and an anhedonia/depression-like deficit (Hikida et al., 2007; Pletnikov et al., 2008; Johnson et al., 2013). In distinction from *DN-DISC1* mice, *N*-ethyl-*N*-nitrosourea (ENU) was used to induce mutations in exon 2 of mouse Disc1 gene, resulting in the occurrence of missense mutations such as Q31L (glutamine to leucine) or L100P (leucine to proline), causing an increase in depression-like behaviors in Q31L mice and schizophrenia-like behaviors including impaired prepulse inhibition (PPI) and latent inhibition in L100P mice (Clapcote et al., 2007; Shoji et al., 2012). Furthermore, the phenotypes of Q31L mutant mice were partly improved with administration of an antidepressant. Thus, DISC1 dysfunction, likely can exert its influence on neuropsychiatric disorders from its role in both neurons and glia.

### An Increase or Decrease of DISC1 Expression in Oligodendrocytes

Expression of Δ*hDISC1* could exert a significant influence on oligodendrocyte proliferation, differentiation, and function (Katsel et al., 2011). In fact, DISC1 is expressed in oligodendrocytes in the corpus callosum (Seshadri et al., 2010). In rat oligodendrocyte precursor cell cultures (Hattori et al., 2014), DISC1 expression decreases in the course of oligodendrocyte differentiation. Furthermore, the expression of CNPase and *myelin basic protein* (MBP) known to markers of myelin maturation were decreased following full length DISC1 overexpression. In contrast, the knockdown of endogenous DISC1 using RNA interference increased the expression of CNPase as well as the number of mature oligodendrocytes (Hattori et al., 2014). SRY box containing (Sox) family member, Sox10, the homeobox containing (Hox) transcription factor Nkx2.2, the basic helix-loop-helix (bHLH) family members Olig1 and Olig2 and the inhibitor of DNA binding (Id) family of proteins Id2 and Id4 have all been shown to be involved in the control of oligodendrocyte differentiation (Nicolay et al., 2007; Emery, 2010). Against this backdrop, it is perhaps not surprising that knockdown of DISC1 increased the expression of Sox10 and/or Nkx2.2, and DISC1 overexpression led to the reduced expression of these transcription factors. These findings are strongly supportive of a role for DISC1 in negatively regulating oligodendrocyte differentiation by acting upstream of Sox10 and/or Nkx2.2 to regulate their transcription (Hattori et al., 2014).

### A Decrease of DBZ Expression in Oligodendrocytes

DISC1 binding zinc finger protein (DBZ), also known as ZNF365 or Su48, is a CNS specific member of the DISC1 interactome and is a novel DISC1 binding protein with a predicted C2H2-type zinc-finger motif and coil domains (Hattori et al., 2007). DBZ regulates neurite outgrowth via the DISC1-DBZ interaction in primary neurons and cultured PC12 cells *in vitro* (Hattori et al., 2007). In DBZ knockout mice, oligodendrocytes displaying an immature structural morphology are more abundant than in control mice and the timing of myelination in the corpus callosum is delayed (Koyama et al., 2013). Although this model implicates DBZ function in oligodendrocyte development and myelination, further studies using more specific models such as oligodendrocyte-specific DBZ knockout mice are needed to elucidate the precise function of DBZ (Shimizu et al., 2014).

### DISC1-Deficiency in Astrocytes

Since the administration of the *N*-methyl-*D*-aspartic acid (NMDA) receptor antagonists phencyclidine and MK-801 induce behaviors that closely resemble those observed in schizophrenic patients, dysfunction of the NMDA receptor is regarded as a particularly strong candidate for being a component of the mechanism of schizophrenia (Javitt and Zukin, 1991; Goff and Coyle, 2001). The stereoisomer D-serine binds to the “glycine site” on the NR1 subunit and it is crucial for the activation of this receptor. D-serine acting as co-agonist at the NMDA receptor is involved in synaptic plasticity (Fossat et al., 2012; Rosenberg et al., 2013), and D-amino acid oxidase (DAAO) degrades the D-serine, modulating D-serine levels and thence NMDA receptor function (Duplantier et al., 2009; Strick et al., 2011). The biosynthesis of D-serine was clarified by the purification and molecular cloning of serine racemase (SR), which transforms L-serine to D-serine, and, interestingly, DISC1 binds to and stabilizes SR (Wolosker et al., 1999a,b; De Miranda et al., 2000). Furthermore, D-serine and SR have been predominantly localized to astrocytes ensheathing synapses, especially in brain regions with enriched NMDA receptors, suggesting that D-serine could be acting as a glial transmitter (Puyal et al., 2006; Williams et al., 2006). In this model of selective and inducible expression of mutant DISC1 in astrocytes, the expression of mutant DISC1 downregulates the level of endogenous DISC1 expression in astrocytes. The disruption of DISC1 binding to SR leads to increased ubiquitination and degradation of SR in astrocytes. The decrease of SR in astrocytes results in diminished production of D-serine in astrocytes. This mouse model displays abnormal behaviors like schizophrenia including sensitivity to an NMDA antagonist; MK-801, in an open field test and pre-pulse inhibition of acoustic startle test, and responds to the ameliorative effects of D-serine (Ma et al., 2013).

## Neuregulin-ErbB Signaling

Neuregulins comprise a large family of widely expressed, alternatively spliced epidermal growth factor (EGF)-like domain-containing proteins that have been strongly implicated in neural development (Corfas et al., 2004; Mei and Xiong, 2008). NRG proteins act by binding to and activating members of the ErbB receptor tyrosine kinase family. After the initial discovery of what came to be known as NRG1, five additional NRG1 homologs (NRG2, NRG3, NRG4, NRG5, and NRG6) have been identified. NRG1 proteins bind only to either ErbB3 or ErbB4 causing a conformational change that promotes receptor dimerization and auophosphorylation, and the subsequent activation of downstream signaling pathways. However, NRG1 does not bind to the ErbB2 receptor, which functions as a co-receptor that heterodimerizes with ErbB3 or ErbB4. Additionally, ErbB4 is known to be able to function as a homodimer. In adult brains, ErbB receptors are widely and differentially expressed. In general, ErbB2 is expressed in most cells, ErbB3 is mainly found in glial populations, and ErbB4 is enriched in neurons. NRG/ErbB signaling has been widely implicated in psychiatric disorders including schizophrenia, bipolar disorder, or depression (Corfas et al., 2004; Mei and Nave, 2014).

### Astrocyte Specific Disruption of synCAM1 Signaling

Synaptic cell adhesion molecule 1 (SynCAM1) is a member of the immunoglobulin (Ig) superfamily, a large group of proteins involved in cell surface recognition (Williams, 1992; Rougon and Hobert, 2003). SynCAM1 plays an important role in CNS developmental processes such as synaptic assembly (Biederer et al., 2002), enhancement of excitatory synaptic transmission (Sara et al., 2005; Fogel et al., 2007), functional presynaptic differentiation (Sara et al., 2005) and the regulation of synapse number and plasticity (Robbins et al., 2010). SynCAM1 is produced in astrocytes and plays a major role in facilitating astrocyte-to-astrocyte and astrocyte-to-neuron adhesive communication (Sandau et al., 2011). SynCAM1 is co-expressed with ErbB4 in astrocytes (Sandau et al., 2011) and is functionally related to ErbB4 receptors (Carpenter, 2003). Mice carrying a dominant-negative form of SynCAM1, specifically targeted to astrocytes, exhibited an attenuation of changes in diurnal rhythm activity. In addition, the locomotor activity in a dark field is increased and it is attenuated with the psychostimulant D, L-amphetamine, and anxiety is reduced in a zero maze test and an acoustic startle paradigm in these mice. These findings imply that these mice could be utilized as a model of neurodevelopmental disorder (Sandau et al., 2012).

### Neuregulin-ErbB4 Receptors Signaling-Deficiency in Oligodendrocytes

Neuregulin 1-ErbB receptor signaling appears to play a critical role in the ontogeny of psychiatric disorders and this hypothesis has been supported by the identification of altered expression levels and/or function of NRG1, ErbB3, and ErbB4 in patients with schizophrenia (Corfas et al., 2004; Silberberg et al., 2006). Moreover, mice with reduced levels of NRG1 or ErbB4 have exhibited behavioral alterations akin to those found in schizophrenia (Gerlai et al., 2000; Golub et al., 2004; Rimer et al., 2005).

Transgenic mice expressing a dominant-negative ErbB4 receptor in oligodendrocytes exhibit thinner myelin and less complex oligodendrocyte morphology and show schizophrenia-like behaviors including high anxiety and an enhanced sensitization to amphetamine. This abnormal response to amphetamine might be due to altered dopamine signaling as aberrant expressions of dopamine transporter (DAT) and dopamine1-like receptor in the cortex, nucleus accumbens, and striatum are evident (Roy et al., 2007).

### Neuregulin-ErbB3 Receptor Signaling-Deficiency in Oligodendrocytes

Neuregulin 1-ErbB signaling plays, at least in part, a critical role in oligodendrocyte development and CNS myelination (Michailov et al., 2004; Taveggia et al., 2005; Chen et al., 2006). In human prefrontal cortex, microarray analyses revealed that the level of ErbB3 was significantly reduced in schizophrenia subjects relative to a normal cohort (Hakak et al., 2001). This decrement was reproduced by another study (Tkachev et al., 2003). Consistent with the results of human studies, mice with selective ErbB3 receptor deletion in oligodendrocytes demonstrate deficits in social interaction and working memory (Makinodan et al., 2012), suggesting that NRG1-ErbB3 signaling in oligodendrocyte might contribute to the pathogenesis of schizophrenia (Makinodan et al., 2012).

## Other Studies

### Mice with Proteolipid Protein Overexpression

*Proteolipid protein* 1, a major protein in CNS myelin (Inoue et al., 1996; Mimault et al., 1999), is regarded as an “adhesive strut” that binds adjacent lamellae of the compacted myelin membrane (Boison et al., 1995).

Transgenic mice harboring extra copies of the myelin PLP1 gene have demonstrated that at the 2 months of age, the myelin was intact with a normally appearing ion channel distribution (Inoue et al., 1996), whereas the conduction velocity in all axonal tracts tested in the CNS was markedly reduced at this age (Tanaka et al., 2009). This observation was supported by subsequent analysis that these mice showed altered neuron–glia interaction with subtle changes in axonal diameters and paranodal structures, leading to schizophrenia-like behaviors including increased anxiety-related behaviors, reduced PPI, spatial learning deficits, and working memory deficits.

### Nogo-A Knockout Mice

Nogo signaling plays a crucial role in restricting axonal regeneration and compensatory fiber growth in the injured adult mammalian CNS (Schwab, 2004; Yiu and He, 2006). The membrane protein Nogo-A, which is predominantly expressed in oligodendrocytes in the adult brain and in neurons mainly during development, is well-known for its role as one of several currently known inhibitors of neurite outgrowth (Huber et al., 2002; Wang et al., 2002). Postmortem and genetic studies have implicated Nogo-A and its choromosomal location in schizophrenia and bipolar disorder (Coon et al., 1998; Novak et al., 2002).

These mice showed sensorimotor deficits, disrupted latent inhibition, and perseverative behaviors. Furthermore, they displayed an enhanced response to systemic amphetamine in an open field test. These behavioral phenotypes might be due to altered monoaminergic transmitter levels in the striatal and limbic regions and/or increased dopamine D2 receptor expression in the identical brain regions. In contrast, adult mice acutely treated with anti-Nogo-A antibodies did not exhibit abnormal behaviors, but showed increased dopamine D2 receptor expression (Willi et al., 2010).

### Selective Overexpression of Heme Oxygenase-1 (HO-1) in Astrocytes

The heme oxygenases (HOs), which are responsible for the degradation of heme to biliverdin/bilirubin, free iron and carbon monoxide (CO), has been strongly implicated in mammalian CNS aging and diseases (Schipper et al., 2009). Mammalian cells express two isoforms such as an inducible isoform, HO-1, and a constitutively active form, HO-2. Specifically, HO-1, encoded by the HMOX1 gene, is a 32-kDa stress protein, and the induction of the glial HMOX1 gene may lead to pathological brain iron deposition, intracellular oxidative damage, and bioenergetic failure in Alzheimer’s disease and other human CNS disorders such as Parkinson’s disease and schizophrenia (Schipper et al., 2009; Brown, 2011). This mice model displayed sensorimotor deficits, increased spontaneous horizontal movements, and stereotypy. Hyperdopaminergic signaling was identified in the striatum and substantia nigra and the associated neurochemical alterations may contribute to these behaviors (Song et al., 2012).

## Conclusion

In conclusion, studies leveraging different animal models for the enhanced biochemical and physiological understanding of mental disorders have moved from strictly targeting the biology of neurons to also include an examination of the glia, especially since glial cells such as oligodendrocytes, astrocytes, and microglia have emerged as critically important modifiers of both CNS development and function. Postmortem brain analyses have clearly indicated that glial cell abnormalities are present in the brains of patients with schizophrenia. However, it remains uncertain whether the dysregulation and symptoms seen are a primary result of alterations in glial cell biology or the deficits in glial function occur as a side product of neuronal dysfunction. Nonetheless, as we have described, continued use of cell-type specific conditional knockout mice will allow us to better dissect how glial cells are implicated in nervous system dysfunction and perhaps illuminate their role in the pathobiology of psychiatric diseases such as schizophrenia.

## Conflict of Interest Statement

The authors declare that the research was conducted in the absence of any commercial or financial relationships that could be construed as a potential conflict of interest.
